# The current epidemiological status of urogenital schistosomiasis among primary school pupils in Katsina State, Nigeria: An imperative for a scale up of water and sanitation initiative and mass administration of medicines with Praziquantel

**DOI:** 10.1371/journal.pntd.0006636

**Published:** 2018-07-06

**Authors:** Tolulope Ebenezer Atalabi, Stephen Dumebi Adoh, Kingsley Marvin Eze

**Affiliations:** Department of Biological Sciences, Faculty of Science, Federal University Dutsin-Ma, Dutsin-Ma, Katsina State, Nigeria; Australian National University, AUSTRALIA

## Abstract

**Introduction:**

Human schistosomiasis, a debilitating and chronic disease, is among a set of 17 neglected tropical infectious diseases of poverty that is currently posing a threat to the wellbeing of 2 billion people in the world. The SHAWN/WASH and MAM programmes in the study area require epidemiological data to enhance their effectiveness. We therefore embarked on this cross-sectional study with the aim of investigating the prevalence, intensity and risk factors of urogenital schistosomiasis.

**Methodology/ Principal findings:**

Interviewed 484 respondents produced terminal urine samples (between 10.00h – 14.00h) which were analyzed with Medi ─Test Combi 10 and centrifuged at 400 r.p.m for 4 minutes using C2 series Centurion Scientific Centrifuge. Eggs of *S*. *haematobium* were identified with their terminal spines using Motic Binocular Microscope. Data were analyzed with Epi Info 7. In this study, the overall prevalence and arithmetic mean intensity of the infection were 8.68% (6.39─ 11.64) and 80.09 (30.92─129.28) eggs per 10ml of urine respectively. Urogenital schistosomiasis was significantly associated with knowledge about the snail host (χ^2^ = 4.23; P = 0.0398); water contact activities (χ^2^ = 25.788; P = 0.0001), gender (χ^2^ = 16.722; P = 0.0001); age (χ^2^ = 9.589; P = 0.0019); economic status of school attended (χ^2^ = 4.869; P = 0.0273); residence distance from open water sources (χ^2^ = 10.546; P = 0.0012); mothers’ occupational (χ^2^ = 6.081; P = 0.0137) and educational status (χ^2^ = 4.139; P = 0.0419).

**Conclusion/ Significance:**

The overall prevalence obtained in this survey shows that the study area was at a low-risk degree of endemicity for urogenital schistosomiasis. Beneath this is a subtle, latent and deadly morbidity-inducing heavy mean intensity of infection, calling for urgent implementation of WHO recommendation that MAM with PZQ be carried out twice for School-Age Children (enrolled or not enrolled) during their primary schooling age (once each at the point of admission and graduation). The criteria for classifying endemic areas for schistosomiasis should also be reviewed to capture the magnitude of mean intensity of infection rather than prevalence only as this may underplay its epidemiological severity.

## Author summary

In 1851, human schistosomiasis was discovered in Egypt by a German surgeon named Theodor Bilharz. Therefore, it is alternatively called ‘bilharziasis’. Being a disease that is closely associated with poverty in the tropics, chiefly the sub-Saharan Africa, Urinary Schistosomiasis is transmitted to humans who source for non-potable water in open and unwholesome water bodies infested with the infective stage of schistosomes which are excreted by *Bulinus globosus*, a freshwater snail and suitable intermediate host. Before now, many endemic foci of the disease have been discovered with many unidentified yet. Meanwhile, the initiative on Sanitation, Hygiene and Water in Nigeria (SHAWN) in the study area is suffering a major setback due to poor data availability despite being due to round up her activities by early 2018. Consequently, we embarked on this survey to determine: the extent at which Urinary Schistosomiasis is prevalent, its severity, and factors that were responsible for its transmission. We found out that the study area was at a low-moderate risk of endemicity for the disease. However, mean intensity of infection was too high. Based on these, a need for Praziquantel is indisputable. Criteria for categorizing areas endemic for the disease should be revised to embrace the novelty of capturing mean intensity of infection.

## Introduction

Human schistosomiasis, though less fatal, but debilitating and chronic in nature, is among a set of 17 neglected tropical infectious diseases of poverty that is currently posing a threat to the wellbeing of 2 billion people in the world [1]. The aetiological agent of urogenital schistosomiasis is the infective stage (cercaria) of *Schistosoma haematobium*, a digenetic trematode plathyhelminth whose intermediate hosts are some species of gastropod snails in the genus *Bulinus* [2,3].

Maturity of *S*. *haematobium* worms takes place at the portal vein of a human host. Subsequently, each male worm carries a female partner in its genaecophoric canal to the veins of the pelvis where the latter lays a very large number of eggs equipped with terminal spines. Consequently, they rupture the endothelial linings of the vesical and urethral walls, leading to granulomatous inflammation, ulceration, pseudopolyposis, and haematuria [4–6]. As a result of its associated morbidities, an estimate of 280,000 people die annually from human schistosomiasis [7].

Currently, sub-Saharan Africa (SSA) is regarded as the poorest part of the world with 73% of its population living on USD 2 per day [8]. This index of abject poverty is of no doubt a key player in the reported astounding 112 million cases of urogenital schistosomiasis (less than a decade ago) out of the 203.15 million (85% of global figure) active cases of human schistosomiasis in the region [9, 10].

Schistosomiasis was reportedly transmitted into the Northern part of Nigeria by invading Fulani herdsmen from upper Nile valley in the ancient times [11]. However, documented evidence was not obtained until 1881when a German immigrant, Gustav Nachtigal, commented on a high incidence of *haematuria* in Borno, a northeastern State of Nigeria close to Lake Chad [12]. In 1963, the first map showing the distribution of schistosomiasis in Nigeria was produced by Professor Cowper [13]. Currently, Nigeria has 29 million cases of schistosomiasis, the highest in SSA, with 101.3 million people living in close proximity to various endemic foci [14].

The endemicity of schistosomiasis was ascertained in Katsina State as far back as 1963 when prevalences of 95% and above 90% were recorded in Katsina and Kankia Local Government Areas [12].

In 2010, UNICEF signed a contribution agreement with the Government of United Kingdom and Northern Ireland to implement Sanitation, Hygiene and Water in Nigeria (SHAWN) between March 2014 and November 2018. Among others, the initiative aims at providing Water, Sanitation and Hygiene (WASH) facilities for 3,500 schools and 1, 200 health centers. The benefit of this initiative is that pupils would have the capacity to promote personal, domestic, and environmental cleanliness. Invariably, schistosomiasis control programme will be enhanced because the risk of contracting the disease will be drastically reduced to the barest minimum. However, only 11 out of 34 LGAs in Katsina State were supported by the SHAWN initiative as at February 2015 [15].

The SHAWN/ WASH initiative *vis a vis* Mass Administration of Medicines (MAM) with Praziquantel (PZQ) in Katsina State require epidemiological data to achieve their aims. Nonetheless, there is limited information in the literature about the current epidemiological status of urogenital schistosomiasis among Primary School pupils in Katsina State. Therefore, we embarked on this study with the aim of investigating the prevalence, intensity and risk factors of urogenital schistosomiasis in the study area.

## Materials and methods

### Study area

**Fig 1:**
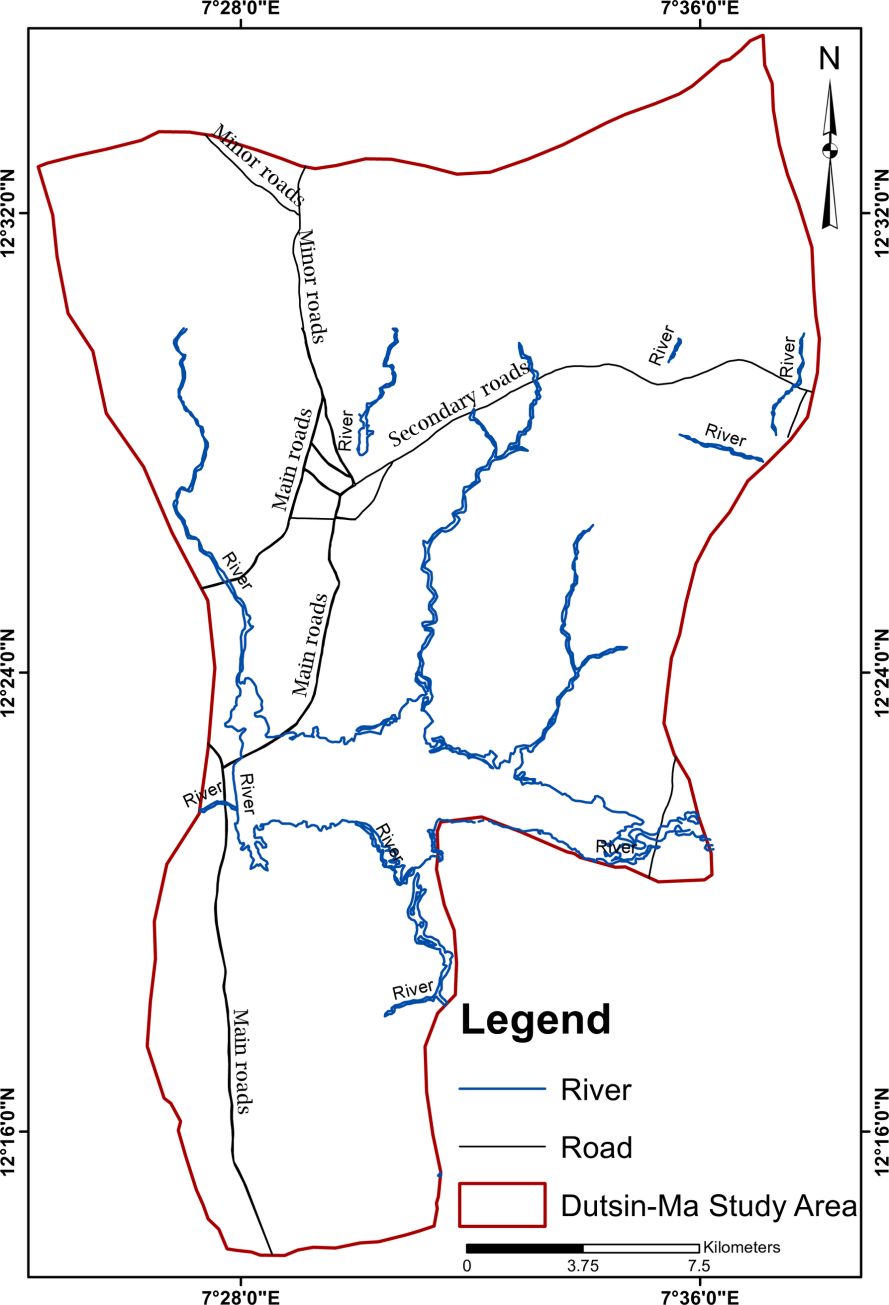
The map of dutsin-ma local government area.

Dutsin-Ma Local Government Area (LGA) [12.45°N and 7.50° E] is located in the eastern part of Katsina State in North western part of Nigeria. It covers a total surface area of 527 km^2^ and is inhabited by a population of 169,829 people as at 2006 National Census [16]. It is predominantly inhabited by Hausa-Fulani tribes whose major occupations are trading, farming, and animal rearing. With a characteristic tropical continental climate type, Dutsin-Ma LGA (see Fig 1) has a temperature range of 29─31°C and mean annual rainfall of 700mm which commences in May and ends by September [17]. As a result of these, its soil is typically sandy and shallow, supporting only cereals like sorghum, millet and grasses. However, short, drought-resistant and scattered trees are present. This vegetation type makes the study area most suitable for pastoralists.

Dutsin-Ma is a rural area characterized with a poor network of healthcare facilities. Generally, potable water is not readily available to average inhabitant of the area. Residents depend on water from unwholesome sources like dams, lakes, and ponds. On the average, a Mono pump, where available, serves over 30 households.

In the study area, MAM with Praziquantel (PZQ) was last carried out in December, 2014.

### Study design

#### Type of study, research population and sample size determination

The study type was a cross-sectional survey carried out between March and July, 2016. This period was most suitable because schools were in session, making pupils available for sampling. Besides, it fell within the rainy season when School-Age Children (SAC) could be observed carrying out water-related activities in unwholesome environments. Prior to sample size determination, we carried out a pilot study in Kandangaru community, where a prevalence of 8.06% was recorded after sampling 62 pupils. The question then arose: “How many pupils should be surveyed so that the overall prevalence anticipated would fall within 4% points (4%─12%) of the true value (8.06%) at 95% confidence level?” We provided answer to this question by using Table 1A on page 25 of sample size estimation recommended by World Health Organization [18]. We found out that a sample size of 216 pupils would be appropriate.

**Table 1:** General characteristics of Nigerian children who participated in the cross-sectional study.

Characteristics	N	(%)	Mean	Standard Deviation
**Gender**				
Males	289	59.71		
Females	195	40.29		
**Age groups (years)**			9.57	2.14
6─10	328	67.77		
11─15	156	32.23		
**Class of school attended**				
Private	127	26.24		
Public	357	73.76		
**Residency duration (years)**				
< 5 years	55	11.36		
≥ 5 years	429	88.64		
**Residence distance from open water source**				
< 100m	192	39.67		
˃ 100m	292	60.33		
**Socioeconomic status**				
**Fathers’ occupation**				
White collar job	153	31.61		
Farming (brown collar job)	36	7.44		
Other brown collar job	295	60.95		
**Mothers’ occupation**				
White collar job	79	16.32		
Other brown collar job	160	33.06		
House wives	245	50.62		
**Fathers’ educational level**				
Tertiary	163	33.68		
Secondary	207	42.77		
Primary	51	10.54		
Illiterate	63	13.02		
**Mothers’ educational level**				
Tertiary	90	18.60		
Secondary	189	39.05		
Primary	79	16.32		
Illiterate	126	26.03		
**Self-report of morbidity indicators**				
Macrohaematuria	73	15.08		
Dysuria	244	50.41		
**History of praziquantel usage**	26	5.37		

Nonetheless, we sampled a total number of 491 pupils because of anticipated factors like effect size, loss of data, voluntary withdrawal, etc. Simple random sampling technique was employed in the enrollment of respondents for the study.

#### Inclusion, exclusion and withdrawal criteria

The study was designed to target Primary School pupils, irrespective of their ages. Only respondents who volunteered were included in the survey. Those who declined to participate were excluded. However, enrolled respondents who failed to submit urine sample after interview were withdrawn from the survey. We had seven (7) cases of withdrawals.

#### Quality control

We ensured universal sample bottles bore corresponding serial numbers to individual questionnaires. Urine samples were promptly analyzed with reagent strips after collection. Dipsticks were read less than 2 minutes of insertion to avoid result falsification. We addressed the tendency for gender bias by involving as many female respondents as were available during the sampling exercise. The average egg count for Urogenital Schistosomiasis in this present survey has been reported as Geometric and Arithmetic Mean Intensities of Infection. The dual advantage here is that the former is more reliable Mathematically while the latter is commonly used by the World Health Organization (WHO) to group the infection into ‘light’ and ‘heavy’ categories. Thus, it enhances interpretation in the light of WHO’s recommendation.

#### Ethical approval

It is noteworthy that there was no formal committee on research ethics in the study area, being a rural setting. Consequently, prior to the commencement of the survey, permission was obtained from the Educational Secretary of the Local Government Education Authority, Dutsin-Ma, Katsina State, Nigeria. Every aspect of the study, including questionnaire and procedure involving sample collection was duly approved. However, identification number was not associated with this approval. Where applicable, a written consent was obtained from the parent/guardian of each pupil with the help of the headmaster. It was not possible to obtain consent from others because the study area has a history of assault/attempted assault on Public Health officials perhaps due to perception that they were on a mission to harm or give them drugs that could render them infertile. In a nutshell, the study area has sensitive peculiarities that require special approach to enhance success of epidemiological surveys. In such case, the school managements gave oral consent after proper briefing on the aim of the study. Pupils were also briefed about the study and offered opportunity to decide whether to participate or not. Consequently, all the pupils enrolled for this survey orally consented to participate. To corroborate this, their names and relevant bio-data were documented using questionnaires. Therefore, informed consent was obtained from parents/guardians/school head of every pupil before prosecuting the study.

#### Questionnaire administration

School-based questionnaire, individual-based questionnaire, & urinalysis forms were used to collect data from respondents. School-based questionnaires were administered to headmasters. It contained data on students’ population, history of Praziquantel distribution, report of haematuria and local languages for haematuria and *Bulinus* species.

Individual-based questionnaire administered to each student contained data on gender, age, sources of water, etc. The knowledge of respondents about *Bulinus globossus* was tested by showing them pictures while being interviewed. The urinalysis form was used to record the result of urine biomedical testing with reagent strips.

### Parasitological procedure

#### Sample collection and analysis using visual test procedure

Terminal urine samples were collected between the hours of 10.00h – 14.00h [19] using sterile corked plastic tubes carrying corresponding labels. The samples were analyzed to obtain chemical parameters like leucocytes, urobilinogen, bilirubin, haematuria, nitrite, pH, specific gravity, proteinuria, glucose, and ketones using Medi ─Test Combi 10 (Duren, German) reagent strip.

#### Urine centrifugation and microscopic examination for *Schistosoma haematobium* eggs

5ml of each urine samples collected was centrifuged at 400 revolutions per minute (r.p.m) for 4 minutes using C2 series Centurion Scientific Centrifuge (United Kingdom). Microscopic examination was performed using x10 objective nose of Motic Binocular Compound Light Microscope (China). Eggs of *S*. *haematobium* were identified with their terminal spines [20]. By using a multiplier factor of 2, intensity of the infection was recorded as number of eggs per 10mls of urine sample and as well categorized into light (< 50 eggs/10 ml of urine) and heavy (≥ 50 eggs/10 ml of urine) infections [21].

#### Data analyses

The data of this survey were entered into Microsoft Excel 2010 (USA). The database was imported to Epi Info 7 (Atlanta, USA) for statistical analyses. The prevalences of urogenital schistosomiasis were calculated by filtering the frequency of the disease with prevalence as a weighing option. However, the geometric mean intensities of urogenital schistosomiasis with their 95% Confidence Interval (CI) were computed using the summary statistics of Small Stata 9.2 (4905, Lake Way Drive, College Station, TX 77845 USA). It became imperative to use the latter because, the former, being a free software, lacked the tool for computing geometric mean. While a bivariate analysis for the relationship between the prevalence of infection and associated determinant factors was performed with Pearson Chi square test, strength of the association was measured using Crude Odds Ratios (CORs) and Risk Ratios with a 95% CI. The p values obtained from Chi Square test were used to screen the variables included in multivariate analysis. By implication, only statistically significant determinant factors were included. Pearson correlation coefficient was estimated for the correlation between determinant factors and prevalence/ intensity of the infection. Odds Ratios obtained in the multivariate analysis were adjusted for possible confounding factors using the logistic regression model.

## Results

### Characteristics of the study population

**Fig 2:**
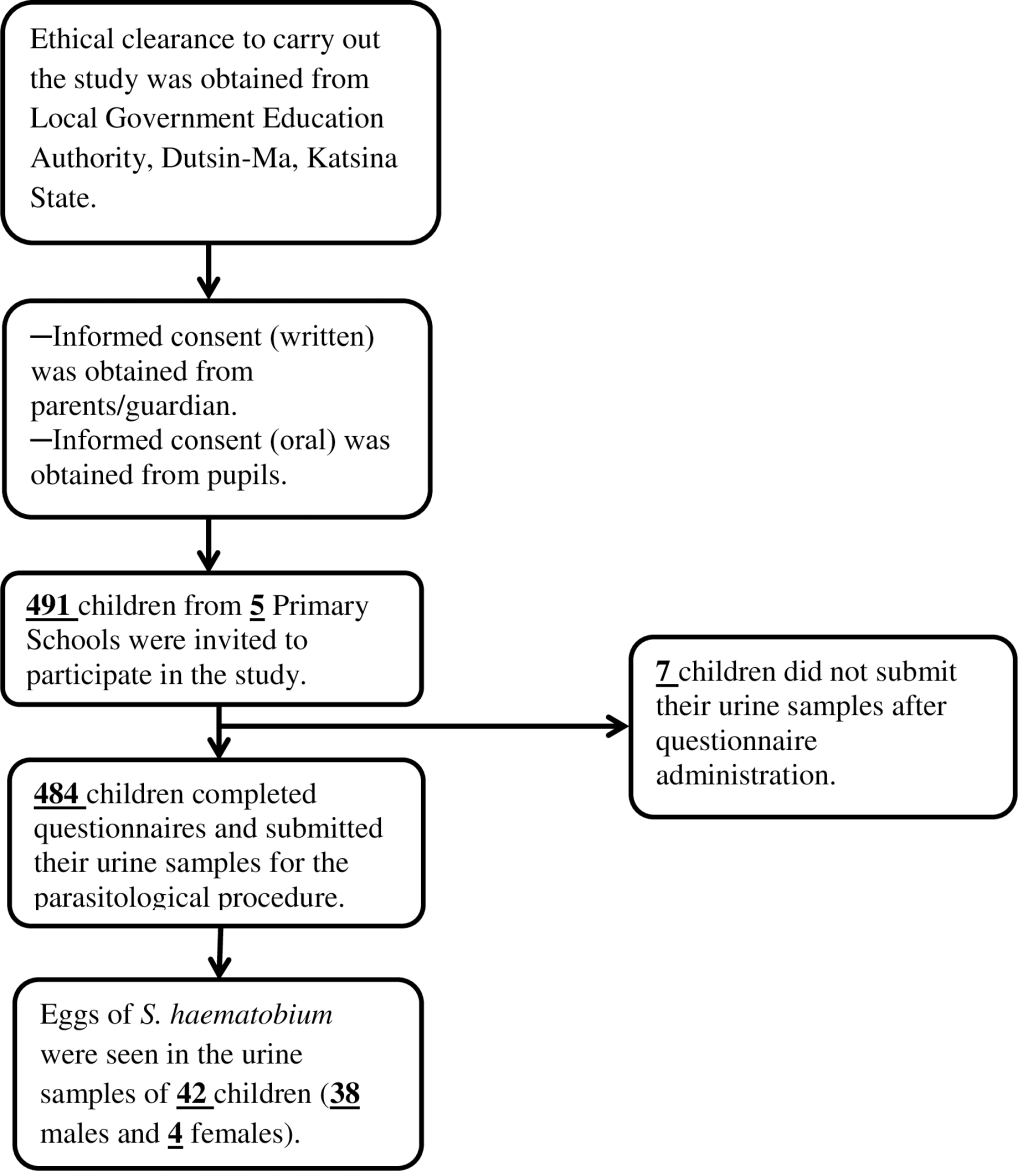
Flow chart of pupils’ participation and compliance in the study.

In this study, a total number of 491 primary school pupils with age range of 6─15 years were recruited from 5 communities in Dutsin-Ma Local Government Area of Katsina State. The mean age ± standard deviation (S.D) was 9.57 ± 2.14 years and 40.29% were females. However, 7 of these pupils did not submit urine samples following interview with questionnaires (see Fig 2). Of noteworthy are the facts that 88.64% had lived in the study area for more than 4 years; 60.33% lived more than 100m away from potentially infested open water sources; only 5.37% had a previous access to Praziquantel while 13.08% and 50.81% reportedly experienced macrohaematuria and dysuria respectively.

Level of illiteracy among mothers of the respondents doubled that of their fathers while access to tertiary education in their fathers almost doubled mothers’. The most common paternal occupational category was “brown collar jobs while mothers of respondents were majorly housewives (see Table 1).

### Knowledge, attitudes and health-seeking practices of respondents in relation to urogenital schistosomiasis

The results about knowledge, attitudes and health-seeking practices of the respondents about urogenital schistosomiasis are shown in Table 2. It was found that 426 (88.02%) and 405 (83.68%), a majority had the awareness of urogenital schistosomiasis (locally called *tsagiyya* or *sarajini*) and the snail intermediate host respectively. Meanwhile, the prevalence of the disease was found to be significantly associated with knowledge about the snail host (χ^2^ = 4.23; P = 0.0398). Of the total number of respondents, 46 (9.50%) had visited health facilities, 18 (3.72%) opted for traditional healing, while 37 (7.64%) received no medication for urogenital schistosomiasis.

**Table 2:** Knowledge, attitudes and health-seeking practices of respondents stratified by school-based prevalence of urogenital schistosomiasis.

Variable	Overall			Private			Public			Chi square	P value
	N	Positive	% (95% CI)	n	Positive	% (95% CI)	n	Positive	% (95% CI)		
**Knowledge**											
Snail host	405	41	10.12 (7.40─13.5)	109	5	4.59 (1.50─10.40)	296	36	12.16 (8.70─16.40)	4.23	0.0398
Macrohaematuria	73	18	24.66 (15.3─36.1)	15	3	20.00 (4.30─48.10)	58	15	25.86 (15.30─39.00)	0.018	0.8938
Dysuria	244	35	14.34 (10.2─19.4)	39	4	10.27 (2.90─24.20)	205	31	15.12 (10.50─20.80)	0.297	0.5855
**Attitude to infection marker**											
It’s a normal thing	58	7	12.07 (5.0─23.3)	10	0	0	48	7	14.58 (6.10─27.80)	─	─
It’s a sign of sickness	426	35	8.22 (5.8─11.2)	117	5	4.27 (1.40─9.70)	309	30	9.71 (6.60─13.60)	2.643	0.1040
**Health-seeking practices**											
Visited health facility	46	7	15.21 (6.30─28.90)	8	1	12.5(0.30─52.70)	38	6	15.79(6.00─31.30)	0.074	0.7596
Visited traditional healer	18	6	33.33 (13.30─59.00)	2	0	0	16	6	37.5(15.20─64.60)	─	─
Self-medication	3	1	33.33 (0.800─90.60)	0	0	0	3	1	33.33(0.80─90.60)	─	─
No medication	37	13	35.14 (20.20─52.50)	2	2	**	35	11	31.43(16.90─49.30)	1.474	0.2247

N, Grand total; n, Sub-total

**, Could not be computed for 95% CI

### Prevalence, intensity and association between urogenital schistosomiasis and its determinant factors

**Fig 3:**
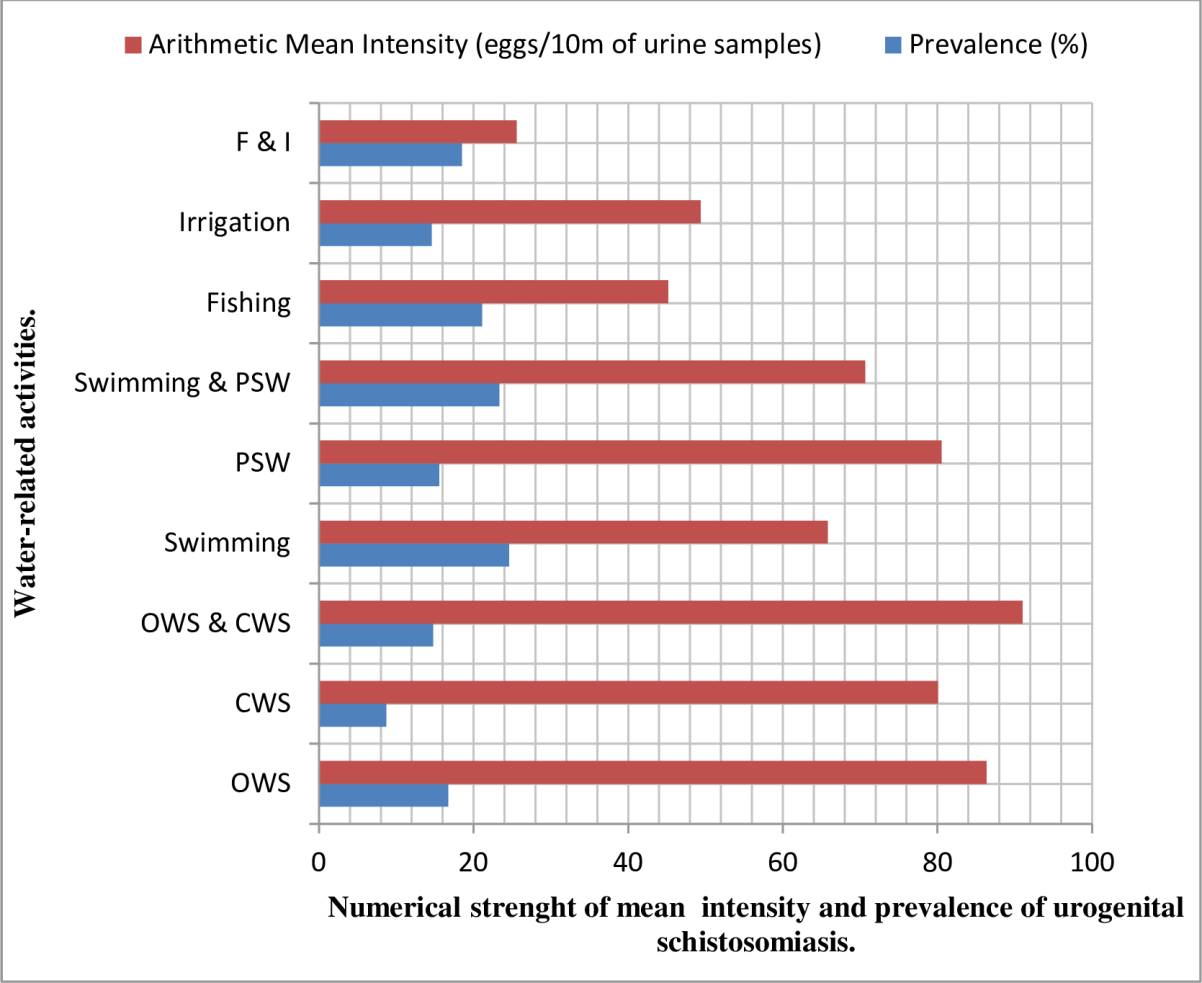
Bar chart showing the arithmetic mean intensity and prevalence of urogenital schistosomiasis with respect to water-related activities of respondents.

The results displayed in Table 3 showed that urogenital schistosomiasis was significantly associated with water contact activities for domestic, recreational and farming purposes (χ^2^ = 25.788; P = 0.0001). Respondents who indulged in the habit of swimming for recreation recorded the highest prevalence of 24.59% (17.20─33.20) while being 3 times more likely to be infected with urogenital schistosomiasis compared with those who only had contact with closed, non-infested water sources [COR (95% CI): 3.39(2.01─5.72)]. Those who combined swimming with play in shallow waters had the second highest prevalence (see Fig 3) of 23.36% (15.70─32.50) and as well 3 times more likely to be infected [COR (95% CI): 3.18(1.82─5.49)]. Nonetheless, those who combined open and closed water sources, against expectation, recorded the highest mean intensity of the infection [27.83(15.78─49.09) eggs/ 10ml of urine sample] followed by respondents with experience of water contact by irrigation [26.92(12.92─56.08) eggs/ 10ml of urine sample].

**Table 3:** Prevalence and mean intensity of urogenital schistosomiasis with respect to water contact for domestic, recreational and farm-related activities.

Variables	N	Positive	Prevalence (95% CI)	OR (95% CI)	EC	GMEC(95% CI)	AMEC(95% CI)
**RDOWS**							
**OWS**	197	33	16.75(11.80─22.70)	2.10(1.28─3.43)	2850	24.66(13.69─44.42)	86.36 (24.48─148.25)
**CWS**	481	42	8.73(6.40─11.60)	1(reference)	3364	26.37(16.30─42.64)	80.09(30.91─129.28)
**OWS & CWS**	230	34	14.78(10.50─20.00)	1.81(1.11─2.94)	3095	27.83(15.78─49.09)	91.03(30.52─151.54)
**Swimming**	122	30	24.59(17.20─33.20)	3.39(2.01─5.72)	1975	25.74(15.46─42.85)	65.83(17.28─114.39)
**PSW**	244	38	15.57(11.00─20.10)	1.93(1.20─3.09)	3060	26.18(16.07─42.65)	80.53(26.32─134.74)
**Swimming & PSW**	107	25	23.36(15.70─32.50)	3.18(1.82─5.49)	1767	25.17(13.82─45.79)	70.68(12.31─129.05)
**Fishing**	71	15	21.13(12.30─32.40)	2.79(1.42─5.31)	678	24.04(13.19─43.84)	45.2(7.05─83.35)
**Irrigation**	89	13	14.61(8.00─23.70)	1.79(0.89─3.44)	642	26.92(12.92─56.08)	49.38(12.28─86.49)
**F & I**	27	5	18.52(6.30─38.10)	2.37(0.76─6.33)	128	21.25(8.97─50.35)	25.6(4.39─46.81)
**Chi square**				25.788			
**P value**				0.0001			

RDOWS, Residence distance from open water source; OWS, Open water sources; CWS, Closed water sources; PSW, Play in shallow water; F & I, Fishing & Irrigation; OR, Odds Ratio; EC, Egg count; GMEC, Geometric Mean of Egg Count; CI, Confidence Interval; AMEC, Arithmetic Mean of Egg Count.

Results of Table 4 convincingly show that gender (χ^2^ = 16.722; P = 0.0001); age (χ^2^ = 9.589; P = 0.0019); economic status of school attended (χ^2^ = 4.869; P = 0.0273); residence distance from open water sources (χ^2^ = 10.546; P = 0.0012); mothers’ occupational (χ^2^ = 6.081; P = 0.0137) and educational status (χ^2^ = 4.139; P = 0.0419) were additional determinant factors of urogenital schistosomiasis in the study area.

**Table 4:** Prevalence and mean intensity of urogenital schistosomiasis based on pupils’ sex, age, residence distance from infested open water, parental educational status and socio-economic demographics.

Variables	N	Positive	Prevalence (95% CI)	Egg count	GMEC (95% CI)	AMEC (95% CI)
Sex						
Male	289	38	90.48 (77.38─ 97.34)	3111	25.75(15.41─43.03)	81.87(27.59─136.15)
Female	195	4	9.52 (2.66─ 22.62)	253	33.01(2.88─378.16)	63.25(-32.43─158.93)
**Chi square**			16.722		33.139	
**P value**			0.0001		0.046	
**Age groups (years)**						
6─10	328	19	45.24 (29.80─61.30)	742	17.86(9.00─35/45)	39.05(13.05─65.05)
11─15	156	23	54.76 (38.70─70.20)	2622	36.37(18.25─72.47)	114(26.03─201.97)
**Chi square**			9.589		390.146	
**P value**			0.0019		0.0002	
**ESSA**						
Private	127	5	11.91 (4.00─25.60)	552	76.19(23.28─249.34)	110.4(-20.72─241.52)
Public	357	37	88.09 (74.40─ 96.00)	2812	22.84(13.58─38.42)	76(21.23─130.77)
**Chi square**			4.869			
**P value**			0.0273			
**RDOWS**						
< 100m	192	27	64.29 (48.03─ 78.45)	1960	25.02(13.49─46.42)	72.59(14.39─130.79)
˃ 100m	292	15	35.71 (21.55─ 51.97)	1404	28.97(12.23─68.62)	93.6(-6.13─193.33)
**Chi square**			10.546		40.794	
**P value**			0.0012		0.1652	
**Fathers’ occupation**						
White collar job	153	9	21.43 (10.30─ 36.81)	1013	42.08 (15.55─113.85)	112.56(-58.41─283.52)
Farming (brown collar job)	36	7	16.67 (6.97─ 31.36)	379	17.24 (3.72─79.86)	54.14(-24.96─133.24)
Other brown collar job	295	26	61.90 (45.64─ 76.43)	1972	25.15 (13.15─48.09)	75.85(15.02─136.67)
**Chi square**			0.566			
**P value**			0.4519			
**Mothers’ occupation**						
White collar job	79	1	2.38 (0.06─ 12.57)	23	23	23
Other brown collar job	160	16	38.10 (23.57─ 54.36)	808	20.20 (8.96─45.57)	50.5(11.51─89.49)
House wives	245	25	59.52 (43.28─ 74.37)	2533	31.43 (16.30─60.61)	101.32(20.84─181.80)
**Chi square**			6.081			
**P value**			0.0137			
**Fathers’ educational level**						
Tertiary	163	14	33.33 (19.57─ 49.55)	1125	30.53 (15.35─60.69)	80.36(-23.84─184.56)
Secondary	207	21	50.00 (34.19─ 65.81)	2171	41.34 (20.99─81.39)	103.38(27.81─178.95)
Primary	51	5	11.90 (3.98─ 25.63)	46	4.68 (0.75─29.11)	9.20(-2.46─ 20.86)
Illiterate	63	2	4.76 (0.58─ 16.16)	22	6.32 (-4.80─8.43)	11(-103.36─125.36)
**Chi square**			1.3151			
**P value**			0.2523			
**Mothers’ educational level**						
Tertiary	90	2	4.76 (0.58─ 16.16)	793	255.15 (0.00─9.46e+07)	396.5(-3459.83─4252.83)
Secondary	189	23	54.76 (38.67─ 70.15)	1829	28.96 (15.52─54.05)	79.52(10.83─148.21)
Primary	79	8	19.05 (8.60─ 34.12)	271	15.19 (4.39─52.49)	33.88(-2.84─70.59)
Illiterate	126	9	21.43 (10.30─ 36.81)	471	20.45 (5.91─70.69)	52.33(-4.73─109.39)
**Chi square**			4.139			
**P value**			0.0419			
**TOTAL**	**484**	**42**	**8.68 (6.39─ 11.64)**	**3364**	**26.37(16.30─42.64)**	**80.09 (30.92─129.28)**

ESSA, Economic status of school attended

In this study, the overall Prevalence and Arithmetic Mean Intensity of the infection were 8.68% (6.39─ 11.64) and 80.09 (30.92─129.28) eggs per 10ml of urine. Of the 42 respondents infected, 27 (64.29%) belonged to the ‘light intensity’ status while 15 (35.71%) fell to the ‘heavy intensity’ category.

Highest values of prevalence were obtained in: males: 90.48% (77.38─ 97.34); age group 11─15: 54.76% (38.70─70.20); public schools: 88.09% (74.40─96.00); respondents whose residence distance are < 100m from open water sources: 64.29% (48.03─78.45); those with brown collar jobs as fathers’ occupation: 61.90% (45.64─76.43); respondents whose mothers were housewives: 59.52% (43.28─74.37); and respondents whose fathers: 50.00% (34.19─ 65.81) and mothers: 54.76% (38.67─70.15) attained secondary level of education.

**Fig 4:**
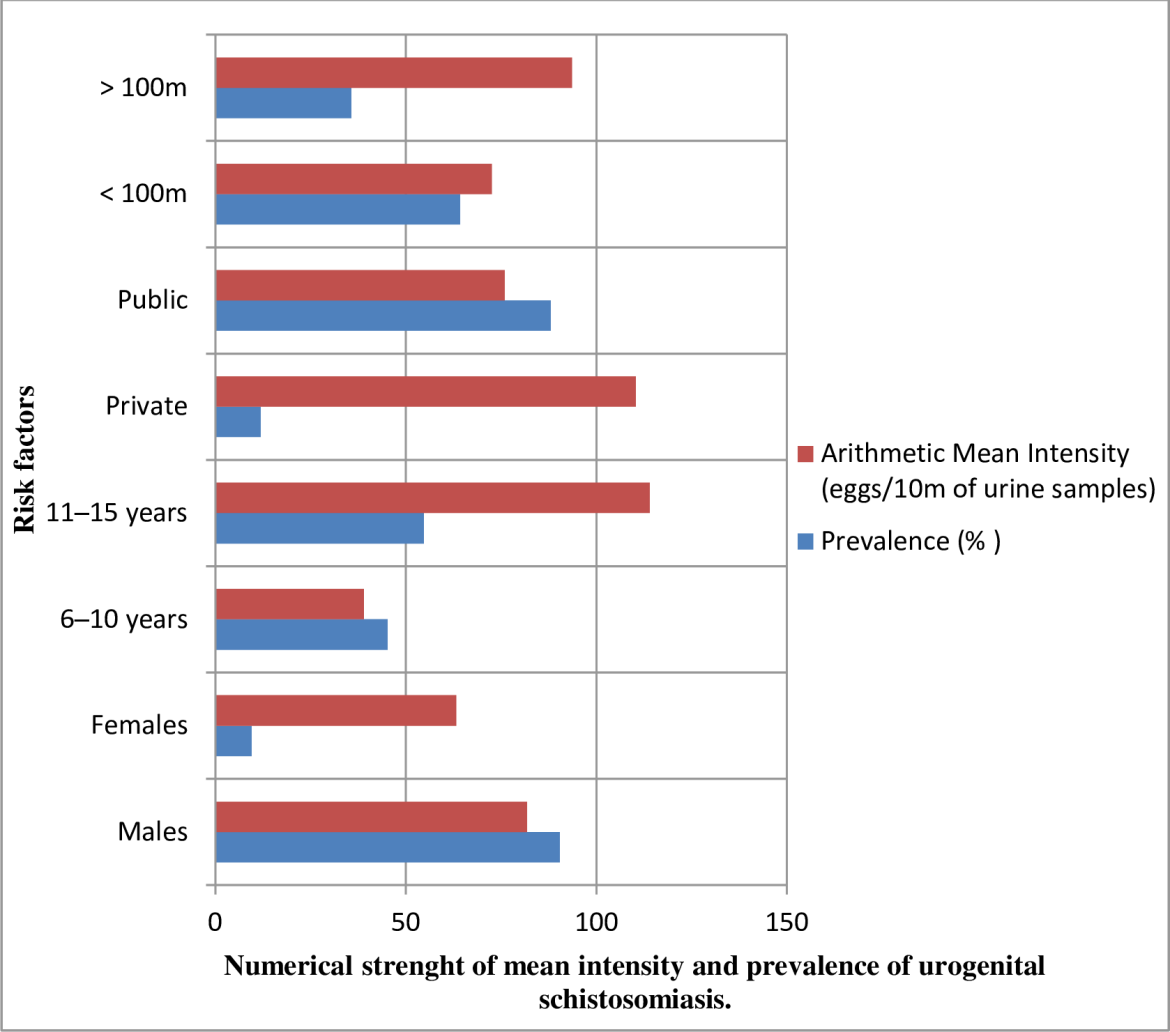
Bar chart showing the arithmetic mean intensity and prevalence of urogenital schistosomiasis with respect to residence distance from open water source, economic status of schools attended, age groups and gender.

Similarly, highest mean intensities of urogenital schistosomiasis were recorded in age group 11─15: 36.37(18.25─72.47) eggs per 10ml (see Fig 4); respondents whose mothers were housewives: 31.43 (16.30─60.61) eggs per 10ml; and respondents whose fathers attained secondary level of education: 41.34 (20.99─81.39) eggs per 10ml. However, contrary to expectation, highest mean intensities of the infection were recorded in females:

33.01(2.88─378.16) eggs per 10ml; private schools: 76.19(23.28─249.34) eggs per 10ml; respondents whose residence distance were ˃ 100m from open water sources: 28.97(12.23─68.62) eggs per 10ml (see Fig 4); those with white collar jobs as fathers’ occupation: 42.08 (15.55─113.85) eggs per 10ml and respondents whose mothers attained tertiary level of education: 255.15 (0.00─9.46e+07) eggs per 10ml.

Table 5 presents data on the strength of association between urogenital schistosomiasis and determinant factors that were found to be significantly associated with it. Male respondents were discovered to be 7 times [AOR (95% CI): 7.23 (2.54–20.60)] more likely to be infected with the cercariae of *S*. *haematobium* compared to their female counterparts. Age group 11–15 years [AOR (95% CI): 2.81 (1.48–5.34)], respondents from public school sector [AOR (95% CI): 2.82(1.15─8.25)] and those living < 100m from open, infested water sources [AOR (95% CI): 3.02(1.56─5.85)] were found to be about 3 times more likely to be infected compared to others in their respective categories.

**Table 5:** Multivariate analysis of the variables associated with the frequency of urogenital schistosomiasis among respondents.

	Frequency of Urogenital Schistosomiasis					
Risk factors	Subtotal	Positive (%)	Negative (%)	Risk ratio (95%CI)	COR (95%CI)	AOR (95% CI)
**Sex**						
Male	289	38(13.15)	251(86.85)	6.41(2.33─17.67)	7.21(2.73─24.08)	7.23(2.54─20.60)
Female	195	4(2.05)	191(97.95)	1	1	1(reference)
**Age groups (years)**						
6─10	328	19(5.79)	309(94.21)	1	1	1(reference)
11─15	156	23(14.74)	133(85.26)	2.55(1.43─4.53)	2.81(1.47─5.39)	2.81(1.48─5.34)
**Class of school attended**						
Private	127	5(3.94)	122(96.06)	1	1	1(reference)
Public	357	37(10.36)	320(89.64)	2.63(1.06─6.55)	2.82(1.15─8.25)	2.82(1.15─8.25)
**Residence distance from open water source**						
< 100m	192	27(14.06)	165(85.94)	2.74(1.49─5.01)	3.01(1.57─5.97)	3.02(1.56─5.85)
˃ 100m	292	15(5.14)	277(94.86)	1	1	1(reference)
**Mothers’ occupation category**						
White Collar Jobs	79	1(1.27)	78(98.73)	1	1	1(reference)
Other Brown Collar Jobs	160	16(10)	144(90)	7.90(1.07─58.51)	8.62(1.51─185.75)	8.66(1.13─66.49)
House Wives	245	25(10.20)	220(89.80)	8.06(1.11─58.54)	8.83(1.61─186.15)	8.85(1.18─66.33)
**Mothers’ educational level**						
Tertiary	90	2(2.22)	88(97.78)	1	1	1(reference)
Secondary	189	23(12.17)	166(87.83)	5.48(1.32─22.72)	6.07(1.62─38.96)	6.09(1.40─26.45)
Primary	79	8(10.13)	71(89.87)	4.56(0.99─20.83)**	4.92(1.09─34.81)	4.95(1.02─24.04)
Illiterate	126	9(7.14)	117(92.86)	3.21(0.71─14.52)**	3.37(0.78─23.37)**	3.38(0.71─16.05)**
**Self-report of morbidity indicators**						
Macrohaematuria	73	18(24.66)	55(75.34)	1.72(1.04─2.85)	1.95(1.01─3.69)	1.94(0.94─3.99)**
Dysuria	244	35(14.34)	209(85.66)	1	1	1(reference)

COR, Crude Odds Ratio; AOR, Adjusted Odds Ratio

**Insignificant association

In Mothers’ occupation category, respondent with “other brown collar jobs” [AOR (95% CI): 8.66(1.13─66.49)] and “housewives” as mothers’ occupation [AOR (95% CI): 8.85(1.18─66.33)] were about 9 times more likely to be infected compared to those whose with “white collar jobs” as mothers’ occupation. It is puzzling that respondents whose mothers attained secondary level of education were 6 times [AOR (95% CI): 6.09(1.40─26.45)] more likely to be infected compared to those whose mothers attained a tertiary education level.

With respect to the risk of infection with *S*. *haematobium*, males who lived in the study area were found to be 6 times more at risk than females [RR (95% CI): 6.41(2.33─17.67)]. In similarity to the odd of infection, age group 11–15 years [RR (95% CI): 2.55(1.43─4.53)], respondents from public school sector [RR (95% CI): 2.63(1.06─6.55)] and those living < 100m from open, infested water sources [RR (95% CI): 2.74(1.49─5.01)] were found to be about 3 times more at risk of being infected compared to others in their respective groups. Respondent with “other brown collar jobs” [RR (95% CI): 7.90(1.07─58.51)] and “housewives” [RR (95% CI): 8.06(1.11─58.54)] as mothers’ occupational categories were about 8 times more at risk of being infected compared to those with “white collar jobs” as mothers’ occupation. The results of our multivariate analysis further showed that subjects whose mothers attained secondary level of education were 5 times [RR (95% CI): 5.48(1.32─22.72)] more at risk of being infected compared to those whose mothers attained a tertiary level of education. Finally, subjects who suffered macrohaematuria were found to be about 2 times [RR (95% CI): 1.72(1.04─2.85)] more at risk of contracting urogenital schoistosomiasis compared to those who experienced dysuria.

## Discussion

In recent years, the World Health Organization recommended five Public Health interventions to speed up the prevention, control, elimination and eradication of NTDs. Our findings will, however, be discussed in the context of three of such interventions that are considered more related: innovative and intensified disease management, provision of safe water, sanitation and hygiene and preventive chemotherapy.

### Innovative and intensified disease management (IDM)

The concept of IDM was devised less than two decades ago to act as a “catalyst” in the control strategies of diseases that are proving difficult to eliminate although effective tools are available. The fundamental principle of this intervention is to ensure that barriers to control strategies are destroyed using the level of awareness about the disease in the population at risk, knowledge of the current epidemiological status of the disease, and the availability and efficacy of available medicines, vaccines and diagnostic tools among others [22].

In the context of this study, knowledge about haematuria and *Bulinus* spp. was used as a strong indicator of awareness about urogenital schistosomiasis. Our findings showed that 426 (88.02%) of the school children surveyed had a knowledge of the disease through the local name for haematuria, *tsagiyya*, while 405 (83.68%), had the awareness of the snail intermediate host. The latter was significantly associated with the disease (see Table 2). The logicality of these findings cannot be subjected to a doubt because infection with the cercariae of *S*. *haematobium* is practically impossible without a transmission cycle involving the suitable snail intermediate host. The strong association clearly suggests that infected respondents most likely came across the snail intermediate host while swimming, bathing, fishing, and/or fetching water in infested streams, rivers, ponds, lakes, and so on. Hunting snails in aquatic terrains might also have been responsible for this. In summary, the cause of this association could be linked to a significant contact (with the snail host versus its aquatic medium) that was enough to encourage transmission of the disease. This knowledge about the snail intermediate host is a strong boost because SAC could be integrated into future control programmes for the disease with the aim of targeting the snails with molluscicides. Besides, physical control measure by handpicking could be enhanced.

Our results on the knowledge of the current epidemiological status of urogenital schistosomiasis showed that the prevalence and arithmetic mean intensity of the disease were 8.68% (6.39─11.64) and 80.09 (30.92─129.28) eggs per 10ml of urine sample respectively (see Table 4). It is obvious that the study area is hypo-endemic (prevalence < 20%) for urogenital schistosomiasis [23] while, based on the arithmetic mean of 80.09 (30.92─129.28) eggs per 10ml of urine sample, an average infected respondent had a heavy intensity of infection. As expected, male respondents had higher prevalence (90.48% Vs 9.52%) and mean intensity of infection (81.87 Vs 63.25).

We also showed through the adjusted Odds Ratio that for every female respondent infected, seven males were infected. Previous studies have reported higher odds of infection with *S*. *haematobium* in male students [24–26].

Meanwhile, previous findings have shown that increase in intensity of infection with urogenital schistosomiasis increased the risk of Female Genital Schistosomiasis (FGS) evidenced by cervical inflammation, intraepithelial neoplasia, postcoital bleeding and genital ulceration [27]. This has been identified as a risk factor in Human Immuno-deficient Virus (HIV) transmission to women [28]. In males, genital schistosomiasis has been reported to induce pathology of the seminal vesicles and the prostate with irreversible long-term consequences which may culminate in bladder cancer, urethral fibrosis and hydronephrosis [28]. Studies have also shown that such high infection intensities make eggs of schistosomes to be trapped in tissues of the liver, spleen and peritonium with severe and complex pathological consequences which may degenerate to late-stage sequelae. Besides, school-age children suffer academic setbacks as well as Iron status deterioration. Organs damaged may not recover until at least six months after cure with Praziquantel [21]. The Risk Ratio obtained from this study revealed that male inhabitants of the study area were six times more at risk of infection with urogenital schistosomiasis (see Table 5). This could be explained by the already known fact that boys are more involved in water contact activities like swimming, fishing, playing in shallow water, irrigation, and making of bricks to build mud houses than girls.

### Efficacy of available preventive chemotherapy and diagnostic tool

Currently, there are no vaccines against *S*. *haematobium*. However, there exist effective diagnostic tools and chemotherapeutic intervention with PZQ, the global drug of choice [21, 29]. Apart from the gold diagnostic standard of microscopy [29–31], a rapid, non-invasive dipstick called Medi-Test, though expensive but readily available in capital cities of Katsina State, was used for the survey as stated earlier. The study area is known for some spurious sentiments about MAM and Public Health survey. Consequently, chemotherapeutic interventions are sometimes seen as attempt to render females infertile. Sometimes, physical attacks with weapons are launched at researchers/ healthcare workers [24]. This probably informed the low PZQ coverage of 5.37% recorded in this survey. As a matter of fact, the record we had on our school-based questionnaires showed that three of the five schools surveyed did not benefit from the previous MAM carried out in 2014. One of the other two who benefitted claimed to have distributed it but response from the pupils during interview negated such claim. It was also likely that it was not done due to the fear of stiff resistance from the mostly uneducated misinformed and indoctrinated parents.

The juxtaposition of this peculiar challenge with the fact that Nigeria ranks number three globally among the countries in most urgent need of chemotherapeutic intervention [22] shows there is still a long way to go to achieving the roadmap for the control of schistosomiasis.

### Provision of safe water, sanitation and hygiene

Water, sanitation and hygiene (WASH) have been identified as part of the key factors determining the state of health in any epidemiological settings [22]. The implication of this is that poor or no access to potable water supply tend to contribute to high prevalence and mean intensity of infection with urogenital schistosomiasis. Our findings in this present study proved this to be true. It showed that respondents who had experience of swimming recorded the highest prevalence, followed by those who combined both the recreational activities of swimming and playing in shallow water. Those who were involved in farm-related activities of fishing had the third placed prevalence. Respondents who combined open, potentially infested with closed water sources recorded the highest mean intensity of the infection, being closely trailed by those who came into contact with open, unwholesome water through the activity of irrigation. Generally, respondents whole came into contact with open water sources like rivers, ponds, streams and lakes were two or three times more likely to be infected with the infective larval form of *S haematobium* (see Table 3) compared to their counterparts who had contact with well, tap water, bore hole and sachet water (closed water sources).

We stated earlier that only 11 out of 34 LGAs in Katsina State were covered by the SHAWN as at February 2015. This report *vis a vis* the data obtained in this present survey (more importantly; the heavy mean intensities of infection) pointed to the fact that the initiative has a long way to go in view of its deadline of November 2018 given to round up its intervention in the Nigerian State.

### Limitations of the survey

Extrapolation of our findings to the whole communities in the study area will amount to painting a wrong epidemiological picture of urogenital schistosomiaisis [23] partly because of its age-sensitivity and focal nature. To corroborate this, previous studies among high school children have identified the study area as being at “moderate-risk” for the disease [24, 25, 32] as opposed to the “low risk” reported in this present study involving primary school pupils.

This study employed urine centrifugation technique for egg concentration due to a lack of polycarbonate filters that would have provided a better picture of mean intensity of infection. Thus, it is likely that the mean intensity of urogenital schistosomiasis in this survey is under-reported here.

### Conclusion

The overall prevalence obtained in this survey shows that the study area was at a low-risk degree of endemicity for urogenital schistosomiasis. Beneath this is the subtle, latent and deadly morbidity-inducing heavy mean intensity of infection which encompassed all determinant (risk) factors identified in this study, thus calling for urgent intervention. Based on these premises, we uphold the recommendation of Crompton & World Health Organization (WHO) on the execution of chemotherapeutic intervention with PZQ twice for School-Age Children (enrolled or not enrolled) during their primary schooling age (once each at the point of admission and graduation) in the study area [33]. We deemed it fit that, as in the case of loiasis, an NTD, the criteria for classifying endemic areas for schistosomiasis be reviewed to capture the magnitude of mean intensity of infection rather than prevalence only as this may underplay its epidemiological severity. While concluding that SHAWN/WASH and MAM programmes in the study area are seriously lagging behind as evidenced in our findings, we strongly recommend that the State Government *vis a vis* Non-Governmental Organizations (NGOs), as a matter of urgency, form a problem-solving alliance to provide water, sanitation and hygiene facilities as well as PZQ with appropriate networking to the grass-root level. Health education should also be carried out at the grass-root level to create awareness about the various factors that predispose people to the infection.

## Supporting information

S1 Checklist STROBE checklist. (DOC)

S2 Checklist Urinary schistosomiasis survey forms. (PDF)

S3 Checklist (XLSX)

S4 Checklist (XLSX)

S5 Checklist (XLSX)
