# Supplementary material for: The current epidemiological status of urogenital schistosomiasis among primary school pupils in Katsina State, Nigeria: An imperative for a scale up of water and sanitation initiative and mass administration of medicines with Praziquantel
Source: PLoS Negl Trop Dis. 2018 Jul 6;12(7):e0006636. doi: 10.1371/journal.pntd.0006636 (PMC6051670; doi:10.1371/journal.pntd.0006636)
Supplement: S1 Checklist — (DOC) [file pntd.0006636.s001.doc]

STROBE Statement—Checklist of items that should be included in reports of ***cross-sectional studies***

|  | Item No | Recommendation | Manuscript  Section |  |  |
| --- | --- | --- | --- | --- | --- |
| **Title and abstract** | 1 | (*a*) Indicate the study’s design with a commonly used term in the title or the abstract | (Background: Paragraph 1) |  |  |
|  |  | (*b*) Provide in the abstract an informative and balanced summary of what was done and what was found | (Methodology/Principal Findings: Paragraph 1) |  |  |
| Introduction | | |  |  |  |
| Background/rationale | 2 | Explain the scientific background and rationale for the investigation being reported | (Background: Paragraphs 3-6) |  |  |
| Objectives | 3 | State specific objectives, including any prespecified hypotheses | (Background: Paragraph 7) |  |  |
| Methods | | |  |  |  |
| Study design | 4 | Present key elements of study design early in the paper | (Materials and Methodology: Paragraph 4) |  |  |
| Setting | 5 | Describe the setting, locations, and relevant dates, including periods of recruitment, exposure, follow-up, and data collection | (Materials and Methodology: Paragraph 1-5) |  |  |
| Participants | 6 | (*a*) Give the eligibility criteria, and the sources and methods of selection of participants | (Materials and Methodology: Paragraph 6) |  |  |
| Variables | 7 | Clearly define all outcomes, exposures, predictors, potential confounders, and effect modifiers. Give diagnostic criteria, if applicable | (Materials and Methodology: Paragraph 4 |  |  |
| Data sources/ measurement | 8* | For each variable of interest, give sources of data and details of methods of assessment (measurement). Describe comparability of assessment methods if there is more than one group | (Materials and Methodology: Paragraph 5) |  |  |
| Bias | 9 | Describe any efforts to address potential sources of bias | (Materials and Methodology: Paragraph 7 |  |  |
| Study size | 10 | Explain how the study size was arrived at | (Materials and Methodology: Paragraph 4) |  |  |
| Quantitative variables | 11 | Explain how quantitative variables were handled in the analyses. If applicable, describe which groupings were chosen and why | (Materials and Methodology: Paragraph 12) |  |  |
| Statistical methods | 12 | (*a*) Describe all statistical methods, including those used to control for confounding | (Materials and Methodology: Paragraph 12) |  |  |
|  |  | (*b*) Describe any methods used to examine subgroups and interactions | (Materials and Methodology: Paragraph 12) |  |  |
|  |  | (*c*) Explain how missing data were addressed | Not Applicable |  |  |
|  |  | (*d*) If applicable, describe analytical methods taking account of sampling strategy | Not Applicable |  |  |
|  |  | (*e*) Describe any sensitivity analyses | Not Applicable |  |  |
| Results | | |  |  |  |
| Participants | 13* | (a) Report numbers of individuals at each stage of study—eg numbers potentially eligible, examined for eligibility, confirmed eligible, included in the study, completing follow-up, and analysed | (Results: Paragraph 1) |  |  |
|  |  | (b) Give reasons for non-participation at each stage | Not Applicable |  |  |
|  |  | (c) Consider use of a flow diagram | (See Figure 1) |  |  |
| Descriptive data | 14* | (a) Give characteristics of study participants (eg demographic, clinical, social) and information on exposures and potential confounders | (Results: Paragraph 1) |  |  |
|  |  | (b) Indicate number of participants with missing data for each variable of interest | Not Applicable |  |  |
| Outcome data | 15* | Report numbers of outcome events or summary measures | (Results: Paragraph 1/2) |  |  |
| Main results | 16 | (*a*) Give unadjusted estimates and, if applicable, confounder-adjusted estimates and their precision (eg, 95% confidence interval). Make clear which confounders were adjusted for and why they were included | (See Tables 3 & 4) |  |  |
|  |  | (*b*) Report category boundaries when continuous variables were categorized | (Results: Paragraph 4) |  |  |
|  |  | (*c*) If relevant, consider translating estimates of relative risk into absolute risk for a meaningful time period | Not Applicable |  |  |
| Other analyses | 17 | Report other analyses done—eg analyses of subgroups and interactions, and sensitivity analyses | (Results: Paragraphs 8-10) |  |  |
| Discussion | | |  |  |  |
| Key results | 18 | Summarise key results with reference to study objectives | Discussion rather based on WHO recommended Health Interventions. |  |  |
| Limitations | 19 | Discuss limitations of the study, taking into account sources of potential bias or imprecision. Discuss both direction and magnitude of any potential bias | (Discussion: Paragraphs 11/12) |  |  |
| Interpretation | 20 | Give a cautious overall interpretation of results considering objectives, limitations, multiplicity of analyses, results from similar studies, and other relevant evidence | (Discussion: Paragraphs 2-10) |  |  |
| Generalisability | 21 | Discuss the generalisability (external validity) of the study results | (Conclusion: Paragraph 1) |  |  |
| Other information | | |  |  |  |
| Funding | 22 | Give the source of funding and the role of the funders for the present study and, if applicable, for the original study on which the present article is based | Removed in compliance to PLoS NTDs submission guidelines. |  |  |

*Give information separately for exposed and unexposed groups.

**Note:** An Explanation and Elaboration article discusses each checklist item and gives methodological background and published examples of transparent reporting. The STROBE checklist is best used in conjunction with this article (freely available on the Web sites of PLoS Medicine at http://www.plosmedicine.org/, Annals of Internal Medicine at http://www.annals.org/, and Epidemiology at http://www.epidem.com/). Information on the STROBE Initiative is available at www.strobe-statement.org.
