# Supplementary material for: The current epidemiological status of urogenital schistosomiasis among primary school pupils in Katsina State, Nigeria: An imperative for a scale up of water and sanitation initiative and mass administration of medicines with Praziquantel
Source: PLoS Negl Trop Dis. 2018 Jul 6;12(7):e0006636. doi: 10.1371/journal.pntd.0006636 (PMC6051670; doi:10.1371/journal.pntd.0006636)
Supplement: S2 Checklist — (PDF) [file pntd.0006636.s002.pdf]

URINARY SCHISTOSOMIASIS FORM A

(SCHOOL COMMUNITY-BASED QUESTIONNAIRE)

State____________________ LGA________________________

School name____
Population___________

Location of school_______________________________

Have you ever received Praziquantel for distribution? Yes [ ] No [ ]

If yes, was there a successful distribution of praziquantel? Yes [ ] No [ ]

If no, why? ____
____

If yes, were there reported side effects? Yes [ ] No [ ]

Do you know any student in this school who reportedly pass blood-stained urine?
Yes [ ] No [ ]

What is the local language for this condition?

____

What is/are the source(s) of water in this school? -
____

Are you familiar with this snail? (picture of *Bulinus* to be shown)

Yes [ ] No [ ]

If yes, what is its local name? _______________________________

URINARY SCHISTOSOMIASIS FORM B (INDIVIDUAL QUESTIONNAIRE)

1a. Name:_____________________________

b. Age:______ c. Sex:______

2a. Source(s) of water supply

(i) Bore hole [ ]

(ii) Dam [ ]

(iii) Lakes [ ]

(iv) Ponds [ ]

(v) River [ ]

(vi) Streams [ ]

(vii) Well [ ]

(viii) Others (Specify)____________________

2b. Purpose of usage: i. Open water _________

ii. Closed water ________

2c. How often do you fetch water from the **open** source(s) per: (i) day [ ]

(ii) week [ ]

(iii) month [ ]

2d. Distance of open water sources:

(i) 100m [ ]

(ii) Above 100m [ ]

3a. Have you swam before? Yes [ ] No [ ]

3b. Visiting times per: i. Week [ ] ii. Month [ ]

3c. Played in a shallow river/pond before?

Yes [ ] No [ ]

3d. History of: Irrigation [ ] Fishing [ ]

3e. History of itching sensation after water contact (3a -3d) Yes [ ] No [ ]

3f. If yes, how many days? _______

4. Have you seen this type of snail (picture of *Bulinus globossus* presented) before?

Yes [ ] No [ ]

5a. Symptoms experienced in the last two weeks (abdominal pain, blood in urine, pain while urinating, diarrhea, coughing, headache, fever & itching) Yes [ ] No [ ]

5b. Diseases experienced in the last two weeks (abdominal problem, Diarhoea, helminthiasis, malaria, respiratory infections, schistosomiasis, & skin diseases) Yes [ ] No [ ]

6. If yes (bood in urine), how have you been treating it?

a. Hospital [ ]

b. Herbalist [ ]

c. No treatment [ ]

7. What is your impression about bloody urine?

a. Normal thing [ ]

b. Spiritual forces [ ]

c. Sign of sickness [ ]

8a. What are the occupations of your parents?

(i) Father **a.** WCJ [ ] b. Farming c. OBCJ [ ]

(ii) Mother **a.** WCJ [ ] b. Farming c. OBCJ [ ] d. House wife [ ]

8b. Parental(F,M) level of education:

(i) Illiterate [ ] [ ]

(ii) Primary [ ] [ ]

(iii) Secondary [ ] [ ]

(iv) Tertiary [ ] [ ]

8c. How long have you been in this town?____________

9a. Have you taken praziquantel before?

Yes [ ] No [ ]

(At this point, the interviewer will show the drug to the respondent to identify).

(i) If yes, how long ago? ________________

(ii) If no, why? _______________

b. History of Severe Adverse Reactions (SARs). Yes [ ] No [ ]

c. If yes, what form was it?

(i) Nausea or vomiting [ ]

(ii) Fainting [ ]

(iii) General feeling of discomfort [ ]

(iv) Skin rash, hives, or itching [ ]

(v) Dizziness [ ]

(vi) Increased sweating [ ]

(vii) Stomach pain [ ]

(viii) Muscle pains or stiffness [ ]

(ix) Others

(Specify)_______________________________.

URINARY SCHISTOSOMIASIS FORM C (URINE ANALYSIS & MICROSCOPY RESULT)

S/N	INTENSITY (eggs/10ml)	BLOOD	URO.	BILIR.	PROTEIN	NITRITE	KET	GLUCO	PH	SPECIFIC G.	LEUCO	COLOUR	TURB.
